# Supplementary material for: Development of a computer-aided design software for the quantitative evaluation of aesthetic damage
Source: PLoS One. 2019 Dec 18;14(12):e0226322. doi: 10.1371/journal.pone.0226322 (PMC6919621; doi:10.1371/journal.pone.0226322)
Supplement: S1 Code site — 1. (ZIP) [file pone.0226322.s001.zip › [EESC_jr][Projeto]Relatorio_07_21/reports/11/2017.03.14/14:38:09/report.pdf]

Preâmbulo

Na data de 12/03/2016 no horário 12:00, o Perito(a) Mestre Rosane Baldasso, Perita Criminal, designado(a)para proceder ao exame pericial solicitado em: Maria  da Silva, do gênero Feminino, nascido(a) em 23/12/1987, ocupação professora, portador(a) do RG 334577855665 SSPSP, cujo endereço Av das Rosas, passa a expor a avaliação de dano estético corporal.

Exame objetivo da lesão

A lesão ora objeto de análise teve como natureza Arma de fogo ocorrido em 10/04/0017. O prejuízo estético consiste em lesão cicatricial consolidada na região: Rosto no lado inferior, direito.

Trata-se de cicatriz Retrátil de tamanho/frequência Única: Complexa de orientação Horizontal de pigmentação Normocrômica medindo 3 centímetro(s) na horizontal, 2 centímetros na vertical e -2 milímetros de Profundidade. De contornos Irregulares. Perceptivel em 15 centímetros de distância nas relações íntimas. Perceptivel em 2 metros nas relações sociais. A lesão tem repercução na vida diária afetiva conforme fotos anteriores e atuais..

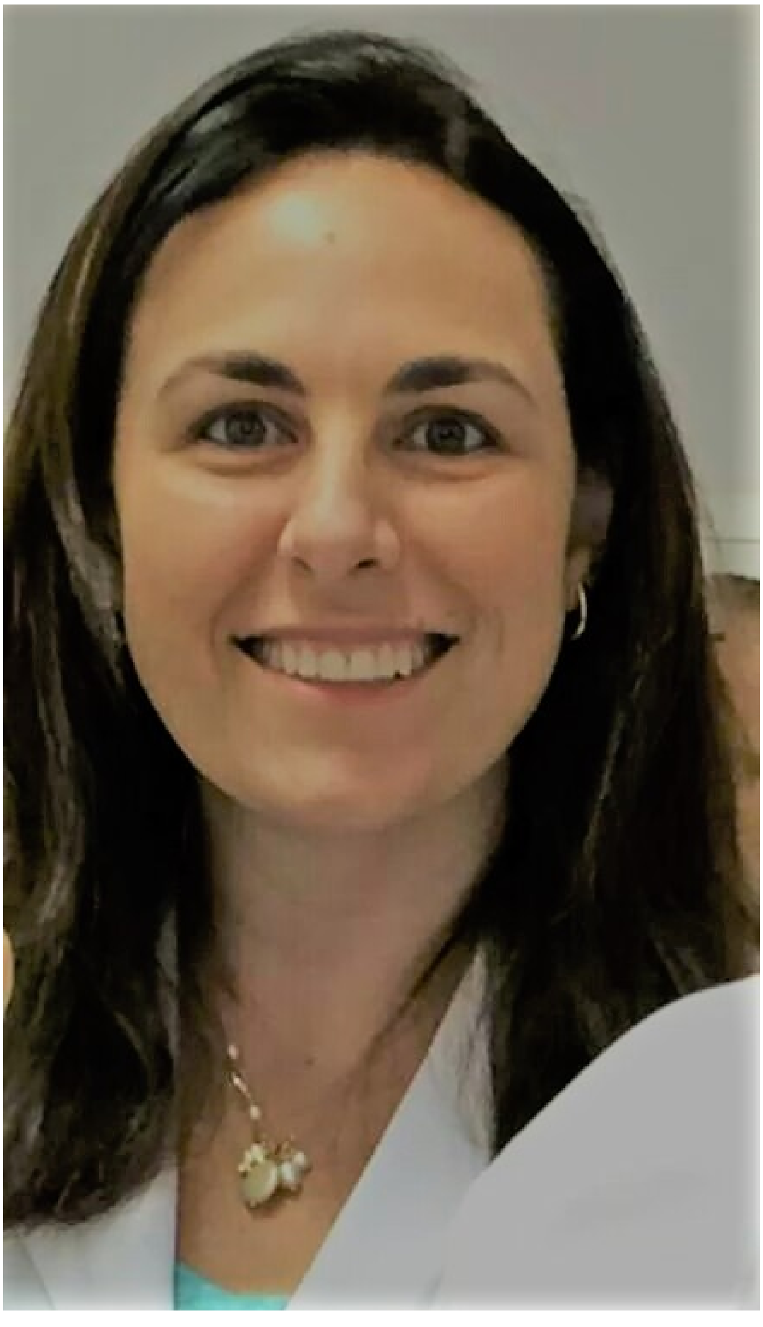

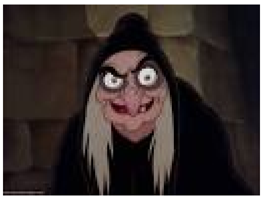

Conclui-se que classificada segundo os parâmetros de dano estético AIPE - Análise do Impacto do Prejuízo Estético (escala de seis níveis e pontuação entre 0 e 50 pontos), como sendo dano estético relativo à área 1, a lesão é caracterizada por 31 pontos e grau de prejuízo estético: Leve.

Escore final: 31;
Grau de prejuízo: Leve.

consultório, 12/03/2016

______________________________

Mestre Rosane Baldasso
